# Permanent defects in renal medullary structure and function after reversal of urinary obstruction

**DOI:** 10.1172/jci.insight.187008

**Published:** 2025-01-23

**Authors:** Thitinee Vanichapol, Alex Gonzalez, Rachel Delgado, Maya Brewer, Kelly A. Clouthier, Anna A. Menshikh, William E. Snyder, Teebro Rahman, Veronika Sander, Haichun Yang, Alan J. Davidson, Mark P. de Caestecker

**Affiliations:** 1 Department of Molecular Medicine and Pathology, University of Auckland, Auckland, New Zealand.; 2 Division of Nephrology, Department of Medicine, and; 3 Department of Pathology, Microbiology and Immunology, Vanderbilt University Medical Center, Nashville, Tennessee, USA.

**Keywords:** Nephrology, Expression profiling, Fibrosis, Urology

## Abstract

Urinary obstruction causes injury to the renal medulla, impairing the ability to concentrate urine and increasing the risk of progressive kidney disease. However, the regenerative capacity of the renal medulla after reversal of obstruction is poorly understood. To investigate this, we developed a mouse model of reversible urinary obstruction. Despite robust regeneration and complete histological recovery of the renal medulla, these mice exhibited a permanent defect in urinary concentrating capacity. However, there were lasting changes in the composition, organization, and transcriptional profiles of epithelial, endothelial, and interstitial cells. Persistent inflammatory responses were also seen in patients with renal stone disease, but there were also adaptive responses to the increasingly hypoxic environment of the renal medulla that occurred only after reversal of obstruction. These findings indicate that while partial repair occurs after reversal of urinary obstruction, there are lasting structural and functional changes across all major cellular compartments of the renal medulla. These changes reflect shared and distinct responses to different renal medullary injuries in humans and mice.

## Abstract 2

Partial repair after reversal of urinary obstruction leads to permanent changes in structure and function of all major cellular compartments in the renal medulla

## Graphical abstract

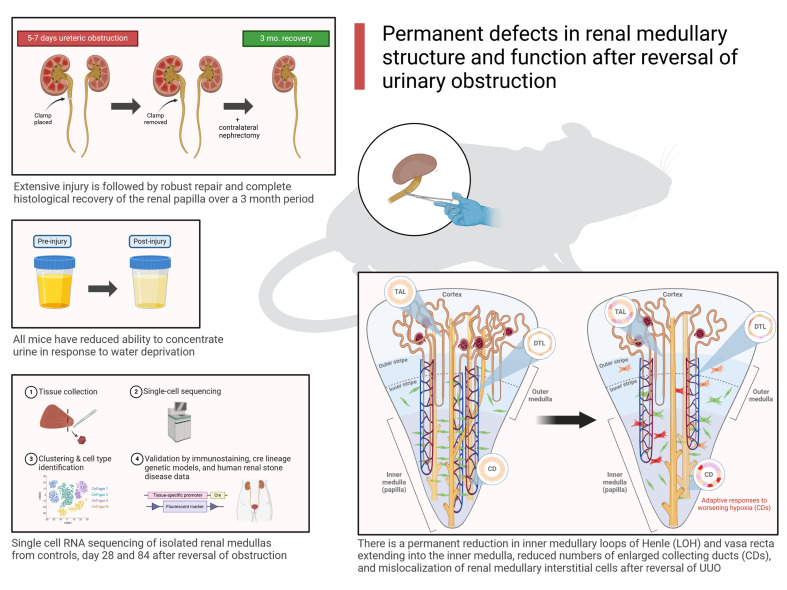

## Introduction

The ability to concentrate urine during water deprivation relies on axial osmotic gradients generated by the renal medulla, which in turn are dependent on maintaining the precise organization of cellular compartments in the inner medulla (IM) (1). This includes the proper arrangement of loops of Henle (LOH), collecting ducts (CDs), and specialized IM capillaries, the vasa recta, which support countercurrent ion and water exchange that is required to generate these osmotic gradients (1, 2). However, while normal IM structure and function are well studied, much less is known about how the IM responds to injury and disease. Urinary obstruction is a common cause of IM injury (3, 4). Treatment involves reversing the obstruction, but patients remain at risk of chronic kidney disease (CKD), and have defects in urine concentrating capacity (UCC), even with early intervention (5–7). Studies in dogs with more than 2 weeks of unilateral ureteric obstruction (UUO) show persistent inability to concentrate urine for up to 18 months after reversal (8). This suggests that obstruction-induced defects in UCC may be permanent. Since failure to concentrate urine predisposes patients to dehydration and recurrent acute kidney injury (AKI), this may contribute to the progressive decline in renal function in these patients.

The renal medulla extends from the corticomedullary junction into the outer medulla (OM) and transitions abruptly from the inner stripe of the outer medulla (ISOM) into the IM. The IM is divided into proximal and distal regions (the latter is also known as the renal papilla) and is characterized by the presence of specialized IM CDs, thin limb (TL) LOH, and vasa recta (9). Urine drains directly from the deep IM CDs into the renal pelvis. The IM is sensitive to damage caused by urinary obstruction, in part due to the increase in pressure in the renal pelvis (10) but also because of reduced IM blood flow (10, 11). As the IM already has low oxygenation (12), this renders it particularly susceptible to ischemic damage after obstruction, which can cause papillary necrosis and radiological evidence of papillary flattening (13, 14). This is recapitulated in mice with irreversible UUO (I-UUO), in which IM compression begins after 3 days of obstruction (15). In rats, 2 weeks of partial ureteric obstruction causes a marked shortening of renal medullas associated with a reduction in the number of IM LOH and CDs (16). However, no studies have evaluated whether these defects are reversible after relief of the obstruction or whether persistent defects in IM structure are sufficient to prevent long-term functional recovery.

Pathophysiological changes that occur after reversal of urinary obstruction in rodents are likely to be similar to those seen in humans because of the highly conserved cellular composition and organization of the renal medulla between the species, despite differences in kidney size and configuration (17). I-UUO models are widely used but fail to mimic the clinical scenario where obstruction is reversed shortly after diagnosis. These models also do not allow the assessment of regenerative responses after reversal. Reversal of ureteric obstruction lasting less than 24 hours in rats shows persistent defects in urine concentration up to 60 days after reversal, linked to downregulation of water channels in the IM LOH and CDs (18, 19). However, these studies do not reflect the impact of prolonged obstruction, which also causes structural damage to the IM. Reversible UUO (R-UUO) models address this but are technically challenging and less commonly used (20). Methods include sequential ureter clamping (20, 21), UUO followed by bladder reimplantation of the ureter (22–25), and use of nondamaging ureteric clamps (26–28). Long-term studies in rats and mice demonstrate improved renal function and reduced cortical and outer medullary fibrosis after R-UUO, though recovery is incomplete if obstruction lasts beyond 2 days (20–23, 26, 28–33). However, aside from short-term studies in rats (18, 19), the long-term effects of reversing prolonged ureteric obstruction on renal medullary structure and function remain unexplored.

Single-cell RNA sequencing (scRNA-Seq) has revealed changes in kidney cell populations before and after AKI (34). Specifically, some proximal tubular epithelial cells (PTECs), which dedifferentiate and proliferate after injury, fail to fully repair and become permanently senescent and pro-inflammatory (35, 36). These “maladaptive” or “failed repair” PTECs (FR-PTECs) are implicated in driving inflammation, fibrosis, and CKD (37–39). FR-PTECs have been identified in various models, including ischemia/reperfusion injury (IRI-AKI) and I-UUO, as well as in human AKI and CKD (35, 36, 40, 41). Single-cell ATAC-Seq studies indicate widespread epigenetic changes in FR-PTECs, which may contribute to their persistent maladaptive and dedifferentiated states (37, 42, 43). However, there are limited data on the fate of IM cell populations in these conditions. One challenge is that IM cells are underrepresented in whole kidney samples used for many scRNA-Seq studies (44). This is important since the cells in the IM are likely to require adaptive responses to survive and recover after injury in the setting of its exceptionally hypoxic and hyperosmolar environment (45).

We present a mouse model of R-UUO in which we evaluated renal function up to 3 months after reversal of prolonged UUO by removing the contralateral kidney. These mice exhibited a permanent reduction in transdermal measurement of glomerular filtration rate (tGFR) and an irreversible defect in UCC. Using scRNA-Seq of isolated renal medullas, validated by cell lineage and quantitative immunostaining studies, we found robust regenerative responses that restored the overall dimensions of the renal papilla after R-UUO. However, permanent alterations occurred across all major cellular compartments in the renal medulla that would be expected to have a profound impact on renal medullary function. Key changes included altered numbers and organization of IM LOH, CDs, and vasa recta, along with reduced and mislocalized renal medullary interstitial cells (RMICs) (46, 47), which regulate UCC (48, 49). Additionally, persistent proinflammatory responses, similar to those seen in FR-PTECs after AKI, were observed in IM LOH and CD cells after R-UUO. These responses were also detected in renal papillary CDs from patients with renal stone disease. These findings suggest a shared chronic injury response to different types of renal medullary damage in humans and mice.

## Results

### Persistent decline in renal function and UCC

**Figure 1:**
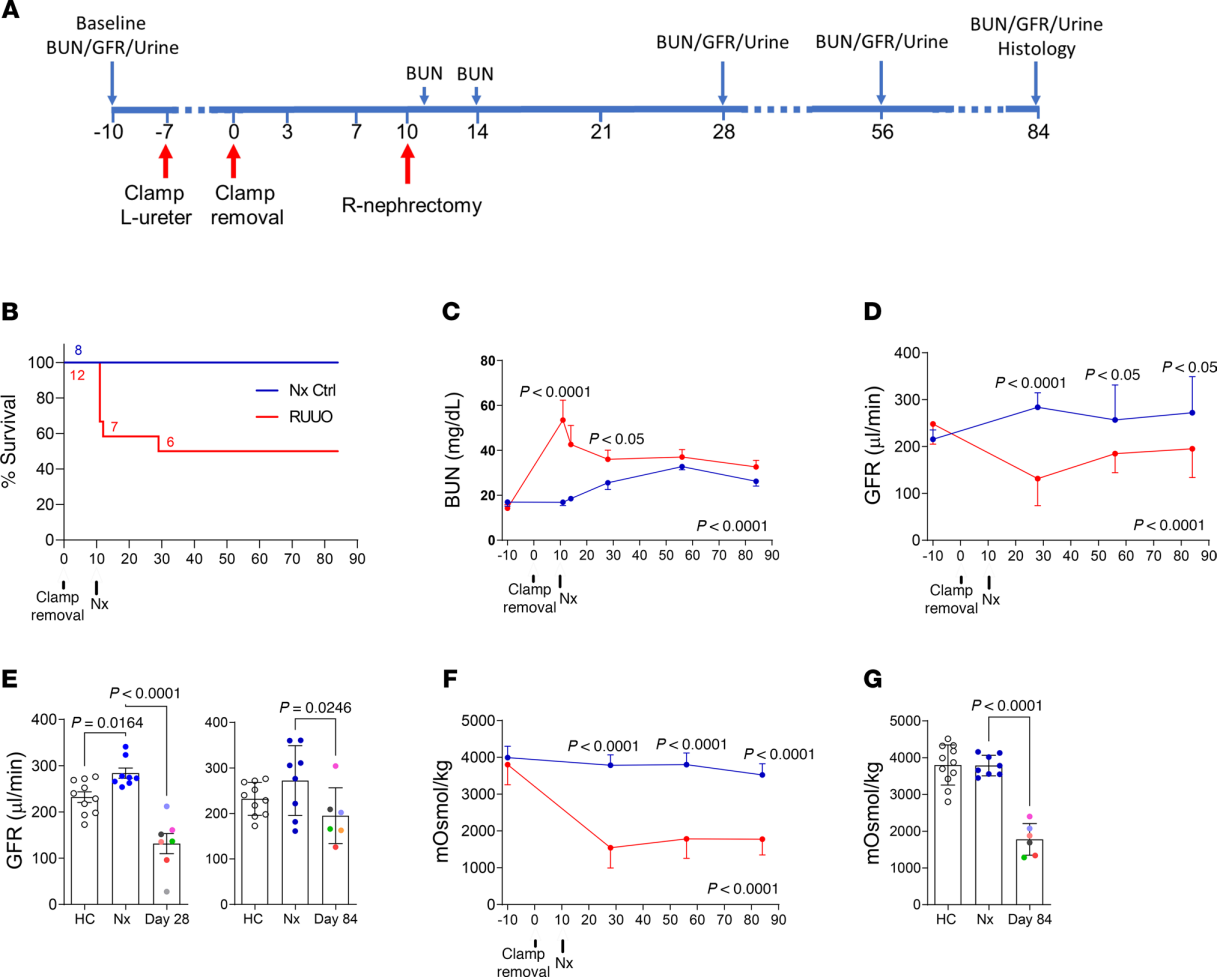
Long-term decrease in renal function and UCC after R-UUO. Male BALB/c mice underwent R-UUO and contralateral Nx or Nx alone. (**A**) Study design; (**B**) survival, with numbers of mice indicated; (**C**) blood urea nitrogen (BUN) time course; (**D**) tGFR time course; (**E**) tGFR at day 28 and 84 after R-UUO. (**F** and **G**) Urinary osmolality after 18-hour water restriction. Time course (**F**) and 84 days after R-UUO (**G**). (**C**, **D**, and **F**) Mean ± SD. Two-way ANOVA. (**E** and **G**) Data points with mean ± SEM. Data points color coded to show relationship between individual tGFR and urine osmolality measurements. One-way ANOVA Nx vs. healthy control and R-UUO. If *P* < 0.05, *q* values for between-group comparisons.

To study the effects of prolonged ureteric obstruction on the kidney, male BALB/c mice underwent R-UUO using a light vascular clamp, followed by contralateral nephrectomy (Nx) 10 days later (Figure 1A and Supplemental Figure 1A; supplemental material available online with this article; https://doi.org/10.1172/jci.insight.187008DS1). In 2 separate experiments, after 7 days of UUO, 30%–50% of mice died within 2 days of Nx, increasing to 80% after 8 days of UUO (Figure 1B and Supplemental Figure 1B). While exclusion of mice dying early after Nx creates a bias for long-term follow-up studies, by performing contralateral Nx 10 days after removing the ureteric clamps, we are excluding those mice in which there is permanent structural or functional loss of ureteric patency resulting in a persistently obstructed kidney. In survivors of 7 days of UUO, blood urea nitrogen (BUN) peaked at ~50–60 mg/dL 1 day after Nx but normalized over time (Figure 1C and Supplemental Figure 1, C and D). There was an increase in serum creatinine at 45 days (Supplemental Figure 1E), and tGFR decreased significantly at 28 and 45 days, improving partially by 84 days but remaining significantly below baseline (Figure 1, D and E, and Supplemental Figure 1F). UCC was markedly reduced at 28, 45, and 84 days after R-UUO (Figure 1, F and G, and Supplemental Figure 1G), indicating lasting renal medullary dysfunction independent of tGFR decline. Survivable UUO clamp times varied between experiments and mouse strains, with 50%–80% survival rates in different experiments (see below). However, all groups showed similar reductions in tGFR and UCC 84 days after R-UUO, suggesting that the severity of functional damage was comparable between strains and experiments.

### Renal medullary injury: early repair and structural remodeling

**Figure 2:**
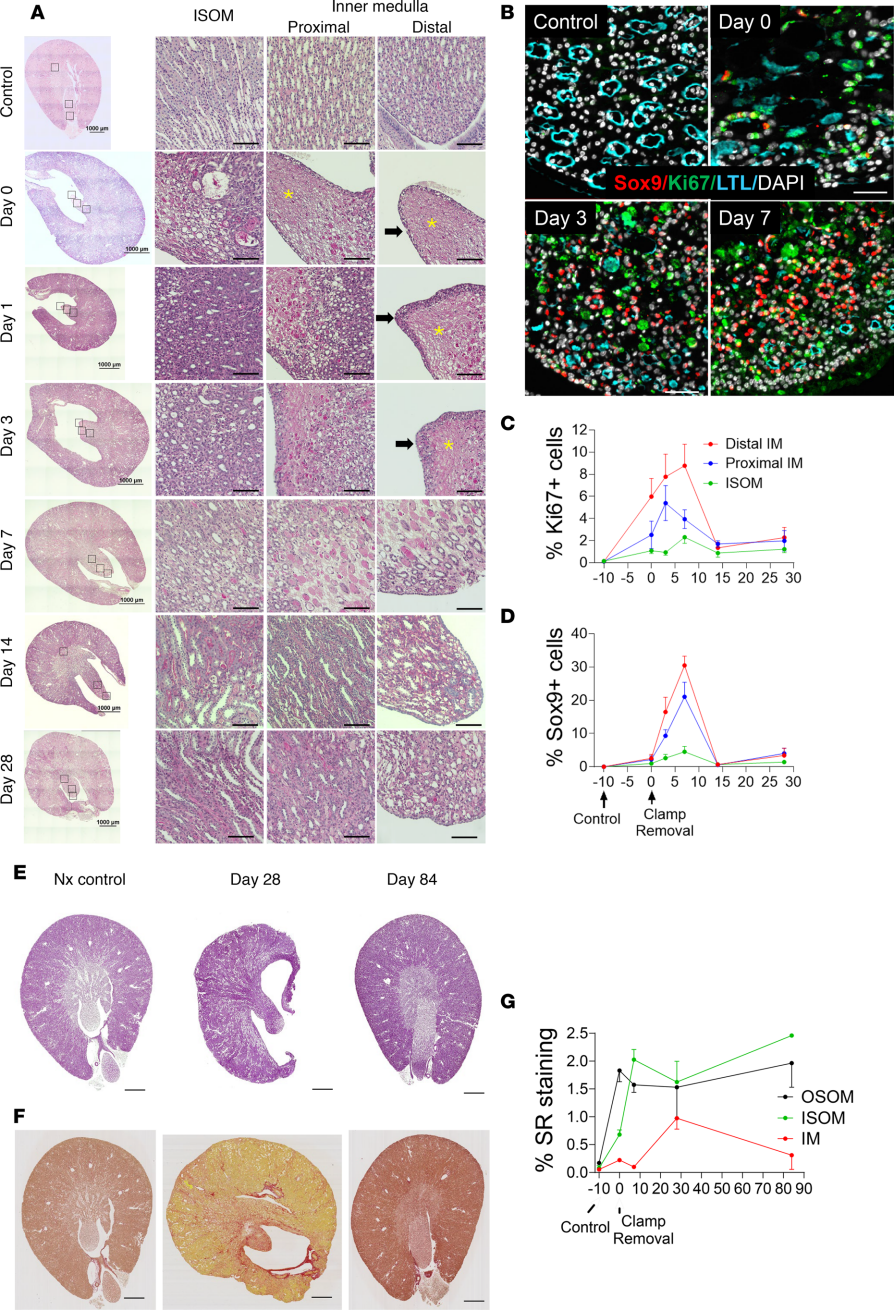
Renal medullary injury, repair, and remodeling after R-UUO. Kidneys were collected at different time points after R-UUO. (**A**) Periodic acid–Schiff (PAS) staining, whole kidney (scale bars = 1 mm), ISOM, and proximal and distal IM (scale bars = 100 μm). *Papillary necrosis; black arrows show thickening of papillary urothelium. (**B**) Ki67, Sox9, and LTL in the distal IM after R-UUO (scale bars = 50 μm). (**C** and **D**) Time course of Ki67^+^ and Sox9^+^ cells (% of total nuclei). *N* = 5-Ctrl, 10-D0, 9-D3, 12-D7, 4-D14, 4-D28. (**E** and **F**) PAS (**E**) and SR (**F**) staining (scale bars = 1 mm). (**G**) Quantification of SR staining. Time course in the OSOM, ISOM, and IM. *N* = 5-Ctrl, 6-D0, 6-D7, 4-D28, 6-D84. Means ± SEM.

Renal histology over 28 days after R-UUO revealed tubular damage and increased peritubular cellularity in the IM (Figure 2A). From day 0 (the time of ureteric clamp release) to 3, more than 50% of mice had papillary necrosis, characterized by denuded tubular basement membranes and loss of nuclei, mostly in the distal IM. Papillary urothelium thickened from 1 to 3 or more cell layers during this period. By day 7, there was evidence of tubular repair, with epithelial repopulation of denuded basement membranes progressing over the next 3 weeks. By day 28, most tubular structures were repopulated, with peaks in cell cycle and regeneration markers (Ki67 and SRY-box transcription factor 9, Sox9) at day 7 (Figure 2, B–D). However, at 28 days, the interstitial space remained disorganized, showing expanded myofibroblast-like cells (Figure 2A and Supplemental Figure 2), but by day 84, the IM recovered its full length and appeared histologically normal (Figure 2E and Supplemental Figure 3). Fibrosis, measured by Sirius red (SR) staining, showed early increase in the OM persisting to day 84. In contrast, SR staining in the IM peaked at 28 days and declined by day 84 (Figure 2, F and G, and Supplemental Figure 4). These findings suggest that while there is robust early repair and remodeling of the IM, subtle defects in IM structure and function, undetectable by standard histology, may underlie the long-term impairment in UCC after R-UUO.

### Permanent loss of AQP1 and transient loss of AQP2 expression in the IM

**Figure 3:**
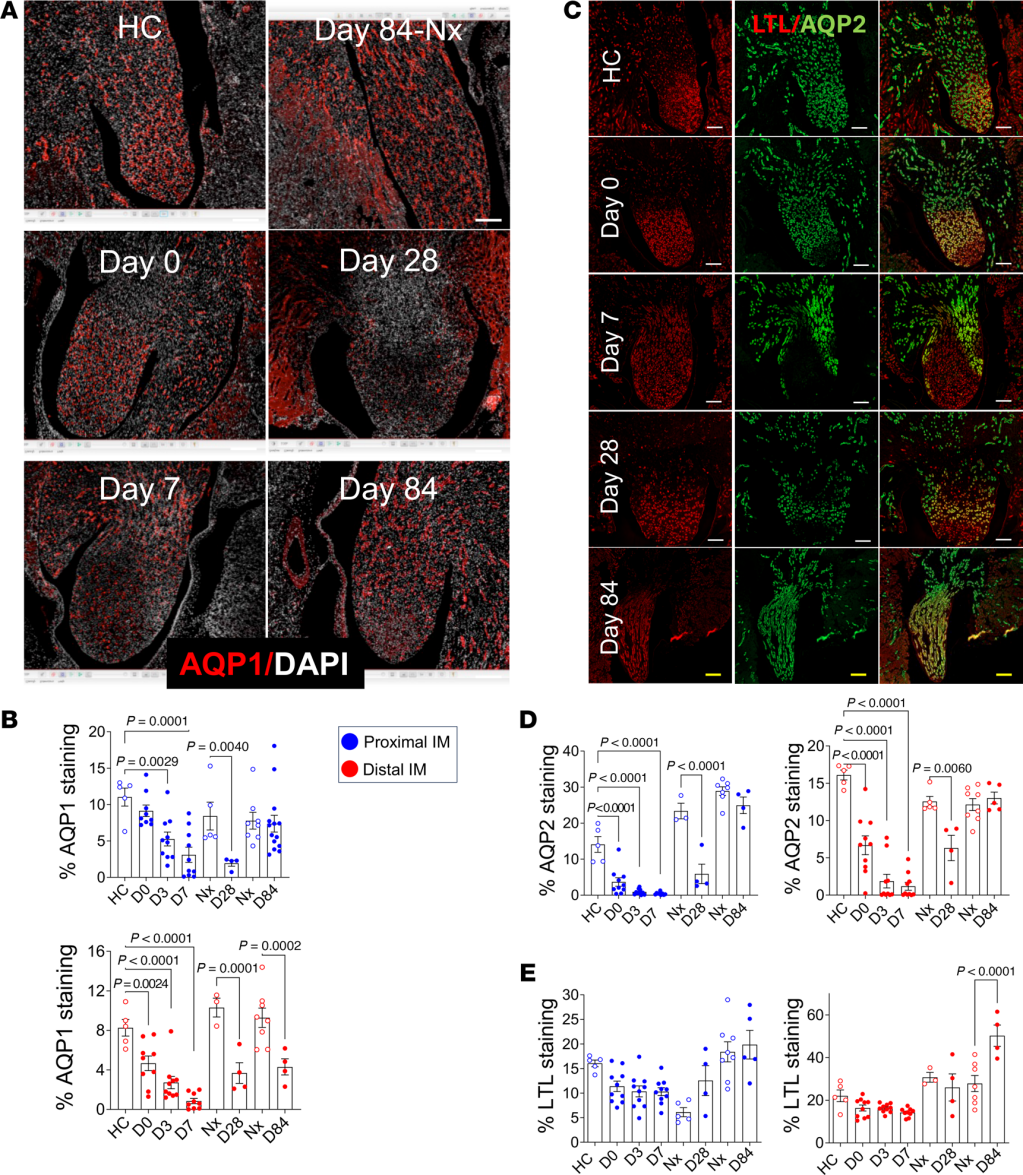
Long-term changes to inner medullary epithelial cells after R-UUO. (**A**) AQP1 staining of the IM after R-UUO (scale bars = 200 μm). (**B**) Quantification of AQP1 staining in the proximal and distal IM time course. Day 28 and 84 Nx shown. (**C**) AQP2 and Lotus Tetroglobinous Lectin (LTL) staining of IM CDs after R-UUO (white scale bars = 200 μm, yellow = 400 μm). (**D** and **E**) Quantification of AQP2 (**D**) and LTL staining (**E**) as the percentage of the area of interest. Data points shown. Blue dots, proximal IM; red dots, distal IM. Means ± SEM. One-way ANOVA, HC or Nx vs. R-UUO time points. If *P* < 0.05, *q* values are shown.

Immunostaining was used to assess markers of differentiated IM descending thin limb (DTL) and CD epithelia. Aquaporin 1 (AQP1), which stains DTLs and descending vasa recta (DVR), showed a significant reduction throughout the IM during the first 7 days after R-UUO (Figure 3, A and B). By day 28, AQP1 expression was still reduced throughout the IM, and at day 84, reduction persisted in the distal but not in the proximal IM. This lasting AQP1 loss may result from fewer differentiated DTLs, reduced LOH extension into the IM, or fewer AQP1-positive DVRs. To evaluate CD epithelia, AQP2, which is expressed by differentiated CD principal cells, was initially reduced throughout the IM for 7 days after R-UUO (Figure 3, C and D). AQP2 staining was still decreased by day 28 but returned to control levels by day 84. In contrast, LTL, which stains IM CDs (Supplemental Figure 5) (50), and V-ATPase–positive intercalated cells (ICs) (Supplemental Figure 6), showed a minor, nonsignificant reduction at early time points but normalized by day 28. By day 84, LTL staining in the distal IM increased significantly (Figure 3, C and E). The restoration of AQP2 expression suggests successful regeneration and differentiation of CD epithelium, while increased LTL staining at day 84 may reflect higher density or surface area of IM CD epithelium or nonspecific staining of other tubular structures.

### Persistent loss of inner medullary cells and expansion of injured cell populations

To assess changes in IM cell populations following R-UUO, scRNA-Seq was performed on dissected renal medullas from BALB/c mice at 28 (*n* = 12) and 84 days (*n* = 6) after R-UUO, compared with healthy controls (*n* = 12). Analysis of 181,255 single-cell sequences identified 7 major cell types (Supplemental Figure 7, A and B). Two technical considerations should be noted, however.

#### Sample collection.

Renal medullas were isolated to enrich IM cell populations as these cells are generally underrepresented in whole kidney scRNA-Seq datasets (44). Immunostaining verified intact IM structures but variable staining of the OM, including ISOM thick ascending limb (TAL; uromodulin staining) and outer stripe of the outer medulla (OSOM) PTECs (LTL staining) (Supplemental Figure 7C). This limits our ability to provide precise quantification of changes in ISOM and OSOM cell populations after R-UUO.

#### scRNA-Seq versus snRNA-Seq.

While single nuclear RNA sequencing (snRNA-Seq) reduces dissociation bias and avoids stress-induced transcriptional artifacts (51), the 10x Genomics fixed-cell RNA-processing method used here offers significant advantages. It employs barcoding for cost-effective, multiplexed sequencing, enabling the analysis of large numbers of singlet cells (43,000–78,000 per pooled sample, Supplemental Figure 7A). This mitigates dissociation bias and enhances the detection of underrepresented cell populations. Additionally, the use of fixed samples prevents stress responses during dissociation, and scRNA-Seq captures both cytoplasmic and nuclear RNA, providing a more comprehensive view of cellular states and gene expression profiles compared with snRNA-Seq.

Using uniform manifold projection (UMAP) plots for visualization and anchor genes to identify cell clusters in the IM versus OM (see Supplemental Methods 1) (44, 52, 53), 25 cell clusters were identified, encompassing IM and OM CD cells, LOH segments, endothelial cells (ECs), fibroblasts, immune cells, and ICs (Supplemental Figure 7, D and E, and Supplemental Table 2). There was a sustained decrease in IM CD, LOH (DTL, ascending thin limb [ATL], and IM TL), and EC populations after R-UUO (Supplemental Figure 7, F and G). Fibroblasts decreased, and myofibroblasts expanded at day 28 and 84 after R-UUO. Populations of injured CD and LOH cells were also detected at day 28 and 84 after R-UUO. These injured cells had reduced expression of classical markers of ATL, DTL, TAL, and CD principal cells and increased expression of injury markers lipocalin 2 (*Lcn2*), secreted phosphoprotein 1 (*Spp1*), *Ccn2*, clusterin (*Clu*), and *Igfbp2* (Supplemental Figure 7, E and H). These injured, partially dedifferentiated cells resemble maladaptive cell populations reported in mice after IRI-AKI and I-UUO and in patients with CKD and sepsis-associated AKI (35, 36, 38–41).

### Sustained reduction of inner medullary LOH

**Figure 4:**
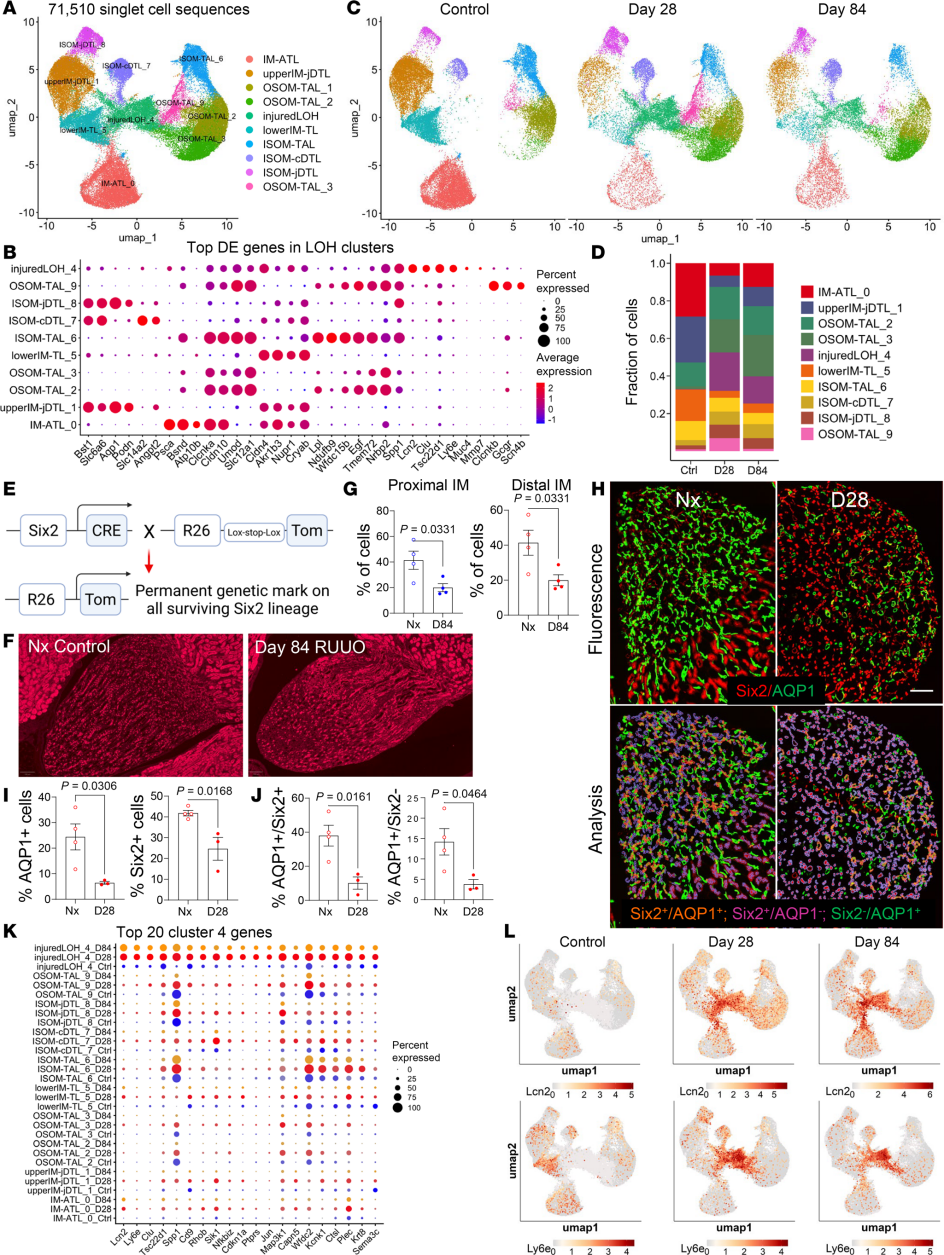
Permanent reduction in inner medullary LOH with expansion of injured cells. (**A** and **C**) Reclustering of LOH cells identified 10 distinct cell populations in the combined dataset (**A**) and after R-UUO (**C**); (**B**) top 5 DEGs for each LOH cluster; (**D**) fractional representation of LOH clusters after R-UUO; (**E**) genetics labeling of IM LOH cells using Six2-Cre mice; (**F**) fluorescence images of Six2 lineage cells in the IM after R-UUO (scale bars = 200 μm); (**G**) quantification of Six2 lineage cells in the IM 84 days after R-UUO; (**H**) fluorescence images with overlays showing AQP1 and Six2 lineage staining in the distal IM 28 days after R-UUO (scale bars = 200 μm); (**I** and **J**) quantification of AQP1^+^ and Six2^+^ cells in the distal IM as the percentage of cells (**I**); AQP1^+^Six2^+^ DTLs (% of Six2^+^ cells), and AQP1^+^Six2^–^ DVRs (% of total cells) 28 days after R-UUO (**J**). Data points, means ± SEM. *P* values determined by *t* test. (**K**) Dot plot of the top 20 DEGs from injured LOH-4 cells in other LOH cell types after R-UUO; (**L**) UMAPs illustrating expression of injury markers *Lcn2* and *Ly6e* after R-UUO.

Reclustering of LOH cells based on differentially expressed genes (DEGs) identified 3 IM TL populations: IM ATL-0, upper IM juxtamedullary nephron DTL (jDTL-1), and lower IM TL-5 cells (Figure 4, A and B, and Supplemental Table 2). All 3 populations showed a marked and persistent reduction 84 days after R-UUO (Figure 4, C and D). Lineage analysis using Six2-Cre R26R-LSL-tdTomato mice, which genetically label nephron epithelium including IM LOH cells but not CDs, validated these findings (Figure 4E). Mice optimized for a 5-day UUO (allowing >50% survival after Nx) exhibited similar long-term reductions in tGFR and UCC to those in the scRNA-Seq studies (Supplemental Figure 8). tdTomato quantification verified a generalized loss of LOH cells throughout the IM 84 days after R-UUO (Figure 4, F and G). To further evaluate LOH cell populations, Six2 lineage markers were combined with AQP1 antibody staining, identifying 3 IM LOH cell types: 1) Six2^+^AQP1^+^ DTL cells, 2) Six2^–^AQP1^+^ DVR cells, and 3) Six2^+^AQP1^–^ ATL and TL cells. Results showed significant reductions in Six2^+^AQP1^+^ DTL cells in the distal IM and Six2^–^ AQP1^+^ DVR cells throughout the IM 28 days after R-UUO (Figure 4, H–J, and Supplemental Figure 9, A–C). However, Six2^+^AQP1^–^ ATL and TL cells showed no change at 28 days (Supplemental Figure 9D). This suggests that the reduction in Six2 lineage cells primarily involves IM DTL cells, while IM ATL and TL populations remain unchanged, contrasting with scRNA-Seq data indicating reductions across all IM LOH populations.

### Expansion and features of injured LOH cell populations

There was an expansion of injured LOH-4 cells after R-UUO. Injured LOH-4 cells exhibit characteristics of failed repair, including partial dedifferentiation and persistent cellular injury. They expressed injury markers (e.g., *Lcn2*, *Spp1*, *Clu*, and *Mmp7*) while showing reduced expression of segment-specific LOH markers (Figure 4B and Supplemental Table 2). Many of their DEGs, such as *Sik1*, *Wfdc2*, *Kcnk1*, and *Krt8*, were either unique to injured LOH-4 cells or also expressed in other LOH clusters in control mice, where they did not change with injury (Figure 4, K and L). Detailed analysis identified 4 subclusters of injured LOH-4 cells (Supplemental Figure 10A). Subclusters 4-1 and 4-3 closely resembled upper IM and ISOM LOH populations (e.g., jDTL-1, ATL-0, TL-5), while subclusters 4-0 and 4-2 were more similar to OSOM and ISOM TAL clusters (e.g., TAL-9 and TAL-5) (Supplemental Figure 10B). UMAP plots for cell type–specific genes, such as *Aqp1* in injured LOH 4-3, and *Psca* in injured LOH 4-0, confirmed the origins of injured LOH-4 subclusters from different LOH cell types in the IM and OM (Supplemental Figure 10, C and D). Gene set enrichment analysis (GSEA) revealed that injured LOH-4 cells were metabolically quiescent, with downregulated pathways for respiration, metabolite processing, and oxidative phosphorylation, but enrichment for cell migration and wound healing processes (Supplemental Figure 11, A and B, and Supplemental Table 3). Hallmark pathways showed increased apoptosis and TNFa via Nfkb signaling. These findings align injured LOH-4 cells with FR-PTECs after I-UUO (40), showing overlap in DEGs and shared pathways for inflammation, migration, and wound healing (Supplemental Figure 12 and Supplemental Table 4). This suggests similar maladaptive repair mechanisms underlie both FR-PTECs and injured LOH-4 cells.

### Minor long-term increase in inflammatory macrophages

Inflammatory macrophages typically expand around failed-repair cells after IRI-AKI (35), but scRNA-Seq revealed only a minor increase in renal medullary inflammatory cells 28 and 84 days after R-UUO (Big UMAP, Supplemental Figure 7, D and F). Reclustering of immune cells identified 3 populations: T cells and 2 macrophage clusters (Mac-0 and Mac-1). T cell numbers decreased, while Mac-1 cells increased after R-UUO (Supplemental Figure 13, A–D, and Supplemental Table 2). GSEA showed activated pathways for migration, adaptive immunity, and phagocytosis in Mac-0 and Mac-1 cells (Supplemental Figure 13E). Hallmark pathways in Mac-1 cells highlighted TNFa via Nfkb and IL-2/STAT signaling, with an overall inflammatory response (Supplemental Figure 13F). However, analysis of pro-inflammatory (M1) and anti-inflammatory (M2) gene sets used to characterize renal macrophage populations after AKI (54–56) revealed no evidence of M1 or M2 polarization in either macrophage cluster (Supplemental Figure 13G). F4/80 antibody staining verified an early increase in macrophages after R-UUO, which decreased over time but remained elevated in the cortex, OM, and proximal IM 84 days after R-UUO (Supplemental Figure 13, H–J). These data indicate that despite the inflammatory signature of injured LOH-4 cells, long-term increases in inflammatory macrophages were modest.

### Permanent reduction in vasa recta throughout the IM

**Figure 5:**
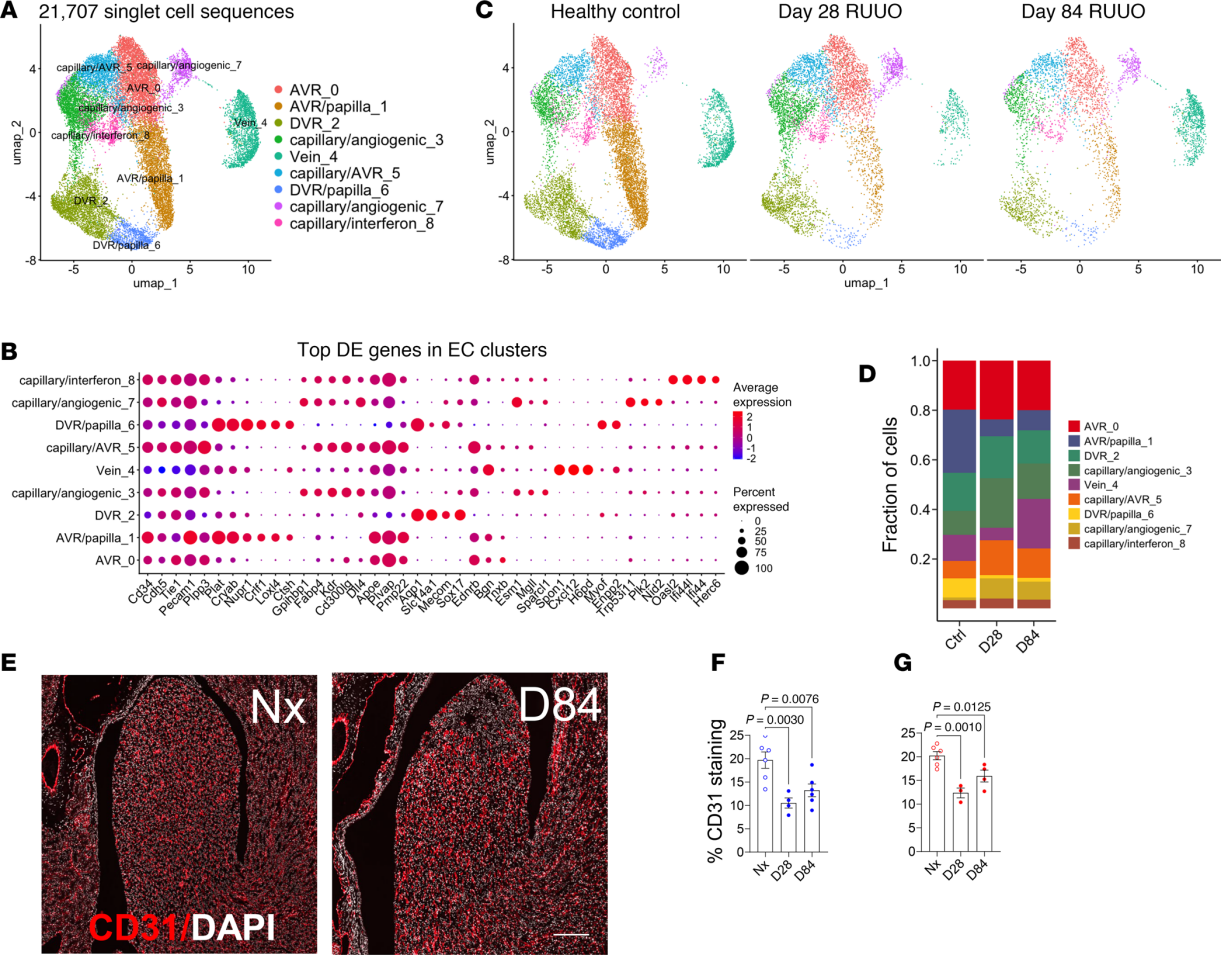
Reduced density of vasa recta in the renal medulla. (**A** and **C**) Reclustering of renal medullary ECs identified 9 populations in the combined dataset (**A**) and at various time points after R-UUO (**C**); (**B**) top 5 DEGs for each EC cluster; (**D**) fractional representation of EC clusters after R-UUO; (**E**) CD31 immunostaining of ECs in the IM 84 days after R-UUO (scale bars = 200 μm); (**F**) quantification of CD31 staining after R-UUO in the proximal (**F**) and distal (**G**) IM. Data are mean ± SEM, analyzed by 1-way ANOVA. *P* < 0.05; *q* values shown where applicable.

Reclustering of ECs identified 9 distinct populations, including ascending vasa recta (AVR)/papilla-1, DVR/papilla-6, AVR-0, DVR-2, and capillary angiogenic-3 and -7 cells (Figure 5, A and B, and Supplemental Table 2). After R-UUO, AVR/papilla-1 and DVR/papilla-6 cell numbers decreased at 28 and 84 days, while capillary angiogenic-3 and -7 cells increased (Figure 5, C and D). GSEA showed enriched Gene Ontology (GO) terms for cell migration in AVR/papilla-1 and DVR/papilla-6 cells, with AVR/papilla-1 cells also displaying ER stress responses (Supplemental Figure 11C). Capillary angiogenic-3 and -7 cells were enriched for angiogenesis and migration pathways, suggesting attempted repair (Supplemental Figure 11D). Despite this, CD31 antibody staining revealed reduced EC density throughout the IM at 84 days after R-UUO (Figure 5, E–G). This is consistent with the reduced Six2 lineage, AQP1^+^ DVR cells and the persistent scRNA-Seq reduction in AVR/papilla-1 and DVR/papilla-6 cells. These findings indicate a permanent loss of IM peritubular capillaries, despite repair efforts. This rarefaction likely contributes to worsening hypoxia, particularly in the distal IM, which may exacerbate cellular injury.

### Temporal and spatial expansion of myofibroblasts

**Figure 6:**
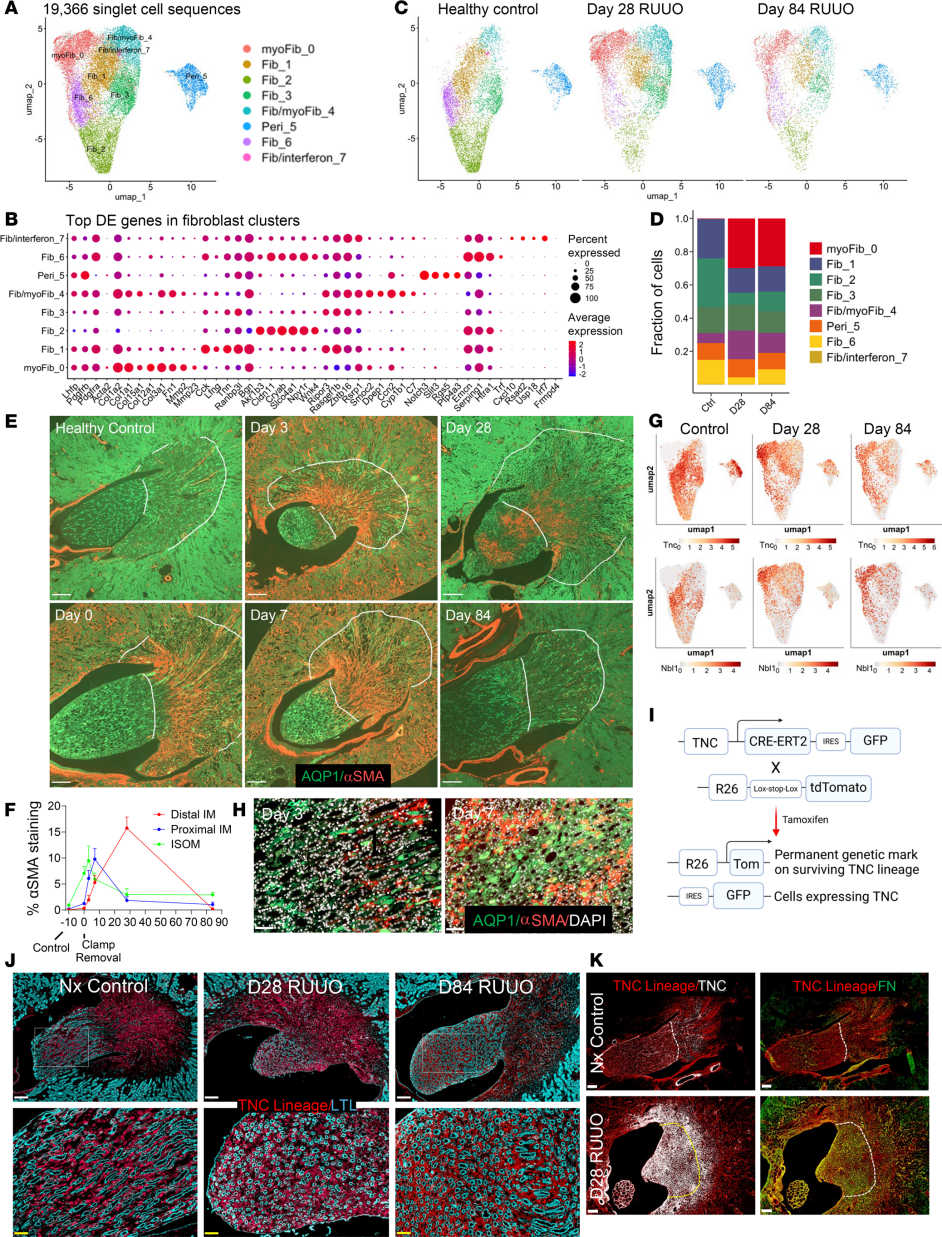
Spatially and temporally restricted myofibroblast expansion derived from RMICs. (**A** and **C**) Reclustering of fibroblasts identified 8 clusters in the combined dataset (**A**) and at various time points after R-UUO (**C**); (**B**) top 5 DEGs for each fibroblast cluster; (**D**) fractional representation of fibroblast populations after R-UUO; (**E**) immunostaining of aSMA and AQP1 after R-UUO (white lines indicating ISOM/IM boundaries, scale bars = 250 μm). (**F**) Quantification of aSMA staining at various time points after R-UUO. *N* = 4-Ctrl, 9-D0, 8-D3, 12-D7, 4-D28, 14-D84. Means ± SEM. (**G**) UMAPs showing RMIC marker distribution (*Tnc* and *Dan/Nbl1*); (**H**) aSMA staining in the distal IM after R-UUO; (**I**) *TNC Cre-ERT2-IRES-GFP* genetics used to track RMIC lineages; (**J**) Immunostaining of Tnc lineage (tdTomato) and LTL (white scale bars = 200 μm, yellow scale bars = 50 μm); (**K**) Tnc lineage (tdTomato) overlaid with GFP to detect active Tnc or FN (dotted lines marking IM/ISOM boundaries, scale bars = 250 μm).

Reclustering of medullary fibroblasts identified 8 subclusters, including 4 fibroblast clusters (Fib-1, Fib-2, Fib-3, Fib-6), a pericyte cluster (Peri-5), and 2 myofibroblast clusters (Fib/Myofib-4, Myofib-0) (Figure 6, A and B, and Supplemental Table 2). After R-UUO, Myofib-0 cells markedly increased, with a smaller increase in Fib/Myofib-4 cells at 28 and 84 days. Concurrently, Fib-2 and Fib-6 cells decreased, Fib-1 cells showed a modest reduction, and no changes were observed in Fib/interferon-7, Fib-3, or Peri-5 cells (Figure 6, C and D). Immunostaining with α–smooth muscle actin (aSMA), a marker for myofibroblasts, revealed increased aSMA^+^ cells in the cortex and OM shortly after R-UUO, peaking in the IM at 28 days (Figure 6, E and F). By day 84, aSMA staining in the ISOM and IM was markedly reduced. Transcriptionally, Myofib-0 cells exhibited low *Acta2* (aSMA) expression but high levels of *Fn1* (fibronectin 1) and type 1 and 3 collagen genes (*Col1a2*, *Col1a1*, *Col3a1*), highlighting their role in extracellular matrix remodeling (Figure 6B and Supplemental Figure 14A). These findings indicate a temporally and spatially restricted expansion of myofibroblasts in response to R-UUO.

### Localization of fibroblast clusters in the renal medulla

Unlike LOH and EC populations, fibroblast anchor genes did not indicate spatial localization within the renal medulla. Hierarchical clustering of fibroblast DEGs showed that Myofib-0 cells were closely related to Fib-2, Fib-6, and Peri-5 cells, while Fib/Myofib-4 and Fib/interferon-7 cells were more related to Fib-1 and Fib-3 cells (Supplemental Figure 14B). *Smoc2* is expressed by Fib/Myofib-4 cells in controls, and in Fib-1, Fib-3, and Myofib-0 cells after R-UUO (Supplemental Figure 14A). Since *Smoc2* is expressed by interstitial fibroblasts in the OM (57), and is increased in OM myofibroblasts after IRI-AKI (58), these findings suggest that Fib-1, Fib-3, and Fib/Myofib-4 cells are likely to be localized in the OM and give rise to OM myofibroblasts. Meanwhile, *Akr1b3*, *Lmo7*, and *CryAB* were expressed in control Fib-2 and Fib-6 clusters and restricted to Fib-2 cells after R-UUO (Supplemental Figure 14A). *Akr1b3* regulates osmolyte synthesis, *Lmo7* is upregulated in hyperosmolar states, and both are increased in the IM (59, 60), but *CryAB* is a molecular chaperone that is increased in all IM cell types (Supplemental Figure 14C) (44). This suggests that Fib-2 and Fib-6 cell clusters are restricted to the IM.

### Renal medullary myofibroblasts derive primarily from RMICs

Expansion of Myofib-0 and Fib/Myofib-4 cells was accompanied by a depletion of Fib-2 and Fib-6, and to a lesser extent Fib-1 cells, but there was no change in vascular pericyte (Peri-5) numbers after R-UUO (Figure 6, C and D). Since pericytes are depleted as they give rise to cortical and OSOM myofibroblasts’ expansion after IRI-AKI and I-UUO (61–63), these findings suggest that Fib-2 and Fib-6, and to a lesser extent Fib-1, contribute to the IM and ISOM myofibroblast expansion after R-UUO, while vascular pericytes play only a minor role. RMICs are specialized renal medullary fibroblasts that express *Tenascin* *C* (*Tnc*) and *Dan/Nbl1* (64) and are also expressed by Fib-1, Fib-2, Fib-3, and Fib-6 cells (Figure 6G). In addition, aSMA staining at early time points after R-UUO showed localized ladder-like patterns characteristic of RMICs (Figure 6H). This suggests that Fib-1, Fib-2, Fib-3, and Fib-6 cells include RMICs and that some of the IM myofibroblasts maybe derived from RMICs. To verify this, we used a tamoxifen-inducible *Tenascin C* *Cre-ERT2-IRES-GFP* mouse model (64). These mice selectively target RMICs but not other fibroblast or pericyte populations in the kidney (48, 49, 64, 65). We evaluated *Tnc* expression using the GFP reporter, and the fate of RMICs by crossing with R26R-LSL-tdTomato reporter mice, which enables tracking RMIC lineage cells after tamoxifen activation (Figure 6I). These mice had 60% survival after 6 days of UUO, and while tGFR was unchanged, UCC was significantly reduced 28 days after R-UUO (Supplemental Figure 15). Tamoxifen-treated mice showed tdTomato^+^ RMIC-derived cells extending throughout the IM and into the ISOM, reaching just below the OSOM/ISOM junction (Figure 6J). However, 28 days after reversal, RMIC lineage cells in the IM appeared disorganized, lacking the characteristic laddered arrangement between tubules (46, 47), and by day 84, there was only partial reorganization of these cells in the IM, since many were no longer arrayed in columns along the length of IM CDs. Costaining with GFP antibodies showed a more limited distribution of *Tnc* expression within the RMIC lineage domains (Figure 6K and Supplemental Figure 16). FN staining, which is limited to a subset of RMIC lineage cells in controls, was expressed by the majority of these cells 28 days after R-UUO (Figure 6K and Supplemental Figure 16). aSMA staining showed a similar distribution to FN, and like FN, a few IM aSMA^+^ cells were *Tnc* lineage negative. FN^+^ and aSMA^+^ myofibroblasts in the renal cortex were also *Tnc* lineage negative. These findings indicate that 1) most myofibroblasts in the ISOM and IM are derived from RMICs; 2) there are at least 2 populations of RMICs, both of which express RMIC markers and contribute to the *Tnc* lineage, including Fib-1 and Fib-3 cells, which also express *Smoc2* and are most likely localized in the OM, and Fib-2 and Fib-6 cells, which express *CryAB*, *Akr1B*, and *Lmo7* and are most likely localized in the IM; and 3) the organization of RMICs in the IM is disrupted as the cells differentiate into myofibroblasts and remains abnormal 84 days after R-UUO.

### Reduced numbers of enlarged CDs in the IM

**Figure 7:**
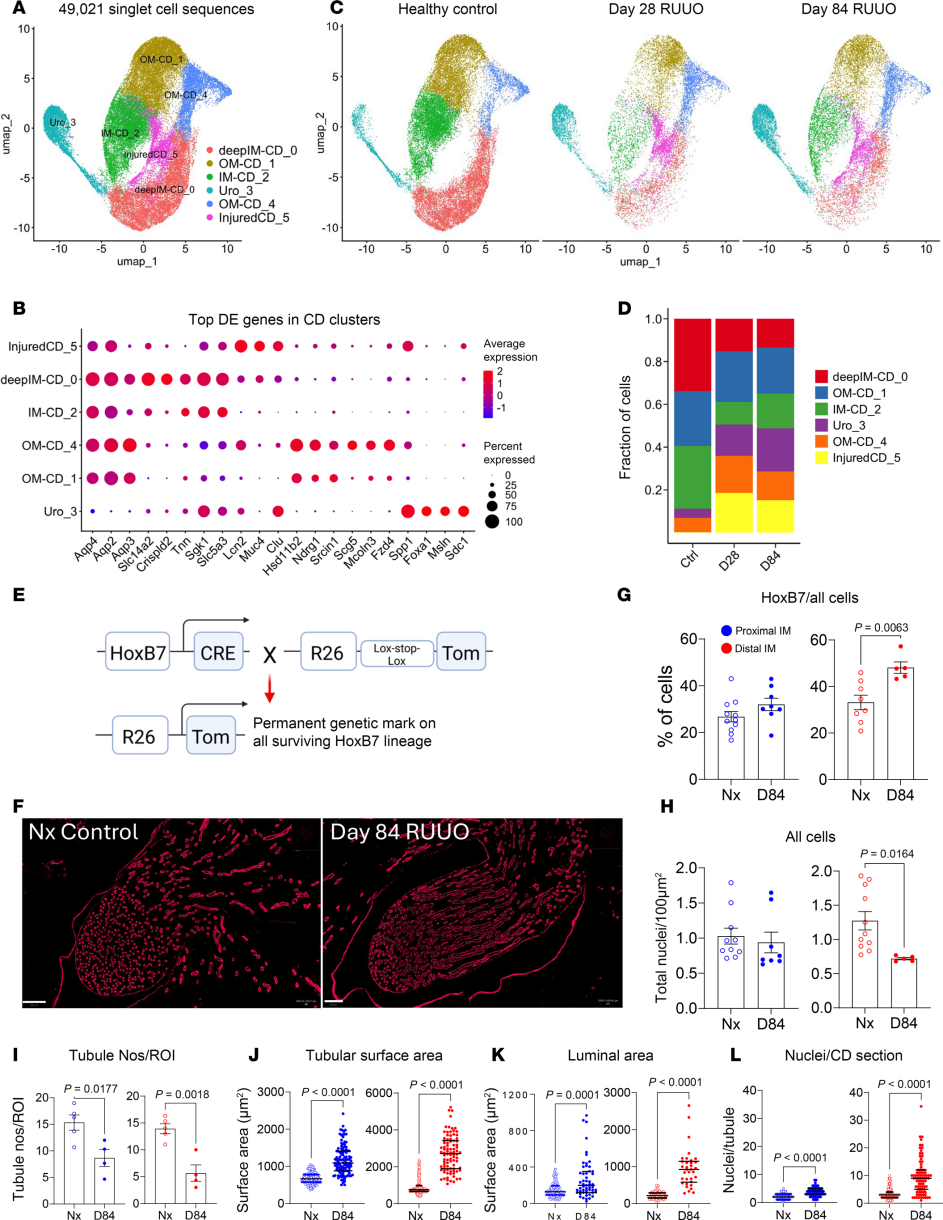
Reduced numbers and enlargement of CDs in the IM after R-UUO. (**A** and **C**) Reclustering of CD principal cells identified 6 populations in the combined dataset (**A**) and at various time points after R-UUO (**C**); (**B**) top 5 DEGs for each CD population; (**D**) fractional representation of CD populations after R-UUO; (**E**) genetic labeling of CD lineage using *HoxB7 Cre*; (**F** and **G**) fluorescence images and quantification of HoxB7 lineage cells 84 days after R-UUO (scale bars = 200 μm); (**I**–**L**) measurement of CD numbers (**I**), surface areas (**J**), luminal areas (**K**), and nuclei per CD section (**L**), using HoxB7-Cre tdTomato and AQP2 staining. Individual data points, means ± SEM. *P* values from *t* tests shown.

Reclustering of CD principal cells revealed 2 CD populations that are spatially restricted to the IM, IM CD-2 and deep IM CD-0 cells (Figure 7, A and B, and Supplemental Table 2). By day 84 after R-UUO, expression of water channels (*Aqp2*, *Aqp3*) and the urea transporter *Slc14a2* were significantly reduced in these cell populations (Supplemental Table 5). Notably, injured CD-5 cells appeared only in R-UUO samples but without significant loss of water channels (*Aqp2*, *3*, *4*, *6*) or urea transporters (*Slc12a1*, *Slc12a2*) compared to other CD clusters. IM CD-2 and deep IM CD-0 cells were also reduced at day 84 after R-UUO (Figure 7, C and D). To investigate further, lineage tracing with *HoxB7 Cre*–knockin mice crossed with R26R-LSL-tdTomato mice was performed (Figure 7, E and F). A 6-day ureteric clamp caused a similar reduction in tGFR and UCC by day 84 as seen in scRNA-Seq studies (Supplemental Figure 17). Quantification showed an increased proportion of HoxB7 lineage CD cells in the distal IM but fewer total cells overall (Figure 7, G and H). This may reflect reduced LOH and EC populations. Detailed analysis revealed fewer CD tubules throughout the IM (Figure 7I) but increased tubule size and luminal surface areas (Figure 7, J and K). Consistent with the increase in proportion of IM HoxB7 lineage cells, the number of nuclei per CD section also rose (Figure 7L). These findings indicate that there is a permanent reduction in IM CD numbers accompanied by compensatory enlargement of the surviving CDs after R-UUO.

### Persistent expression of injury markers in inner medullary CDs after R-UUO

**Figure 8:**
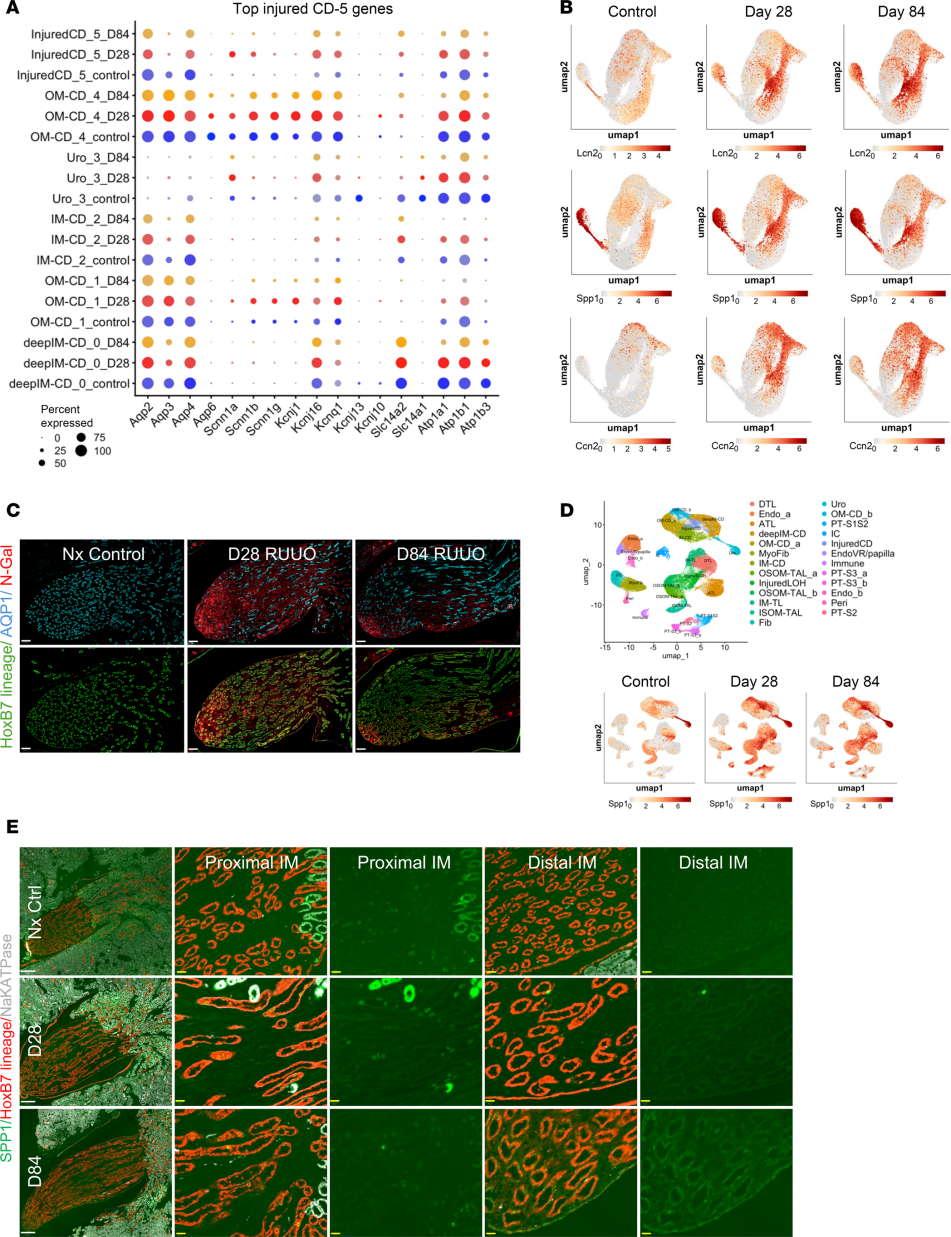
Persistent expression of injury markers in inner medullary CDs. (**A**) Dot plot of the top 25 DEGs from injured CD-5 cells in other CD populations after R-UUO; (**B** and **D**) UMAPs showing the expression of injury markers (*Lcn2*, *Spp1*, *Ccn2*) in CD clusters (**B**) and *Spp1* across all renal medullary single-cell clusters (**D**). (**C**) NGAL, AQP1, and HoxB7 lineage (tdTomato) staining after R-UUO (scale bars = 100 μm); (**E**) SPP1, Na^+^/K^+^-ATPase, and HoxB7 lineage staining after R-UUO (white scale bars = 250 μm, yellow = 50 μm).

Injured CD-5 cells displayed increased expression of injury markers, such as *Lcn2*, *Spp1*, *Clu*, *Mmp7*, and *Ccn1* (Figure 7B and Supplemental Figure 18, A and B). Injured CD-5 cells expressed CD markers like *Aqp2*, *3*, and *4*, found across CD clusters, and *Slc14a2*, typically dominant in deep IM CD-0 cells, suggesting these cells originated from both IM and OM CD populations (Supplemental Figure 18A). To characterize these cells further, we analyzed the top 25 upregulated DEGs in injured CD-5 cells (Figure 8A). Some DEGs, such as *Pdgf* and *Dcdc2a*, were uniquely expressed in CD-5 cells, while others, such as *Clu*, *Mmp7*, *Lcn2*, *Spp1*, and *Gdf15*, were shared with other CD clusters (Figure 8, A and B, and Supplemental Figure 18B). Immunostaining revealed the spatial distribution of 2 injury markers, NGAL (encoded by *Lcn2*) and SPP1, in *HoxB7* *Cre* lineage mice. NGAL was absent in controls but markedly increased after R-UUO in urothelium, AQP1^+^ DTLs, and HoxB7 lineage CD cells in the IM (Figure 8C). Big UMAP analysis showed *Spp1* expression in ISOM TAL, urothelium, and intercalated cells (ICs) in uninjured mice, with increased expression in ICs, urothelium, injured CD cells, deep IM CD cells, ISOM TAL, and injured LOH cells after R-UUO (Figure 8D). Consistently, immunostaining detected SPP1 in TALs (Na^+^/K^+^-ATPase high) and in interspersed HoxB7 lineage cells in the ISOM and proximal IM of uninjured mice (Figure 8E). These cells, likely ICs, were localized in ISOM and proximal IM CDs. After R-UUO, SPP1 staining increased in TALs and presumed ICs by day 28 and in urothelium and distal IM HoxB7 lineage CDs by day 84 (Figure 8E). These findings corroborate scRNA-Seq data and highlight the widespread, persistent expression of injury markers, particularly in the distal IM, after R-UUO.

### Injured inner medullary CDs exhibit hypoxic adaptation and features of failed-repair cells

**Figure 9:**
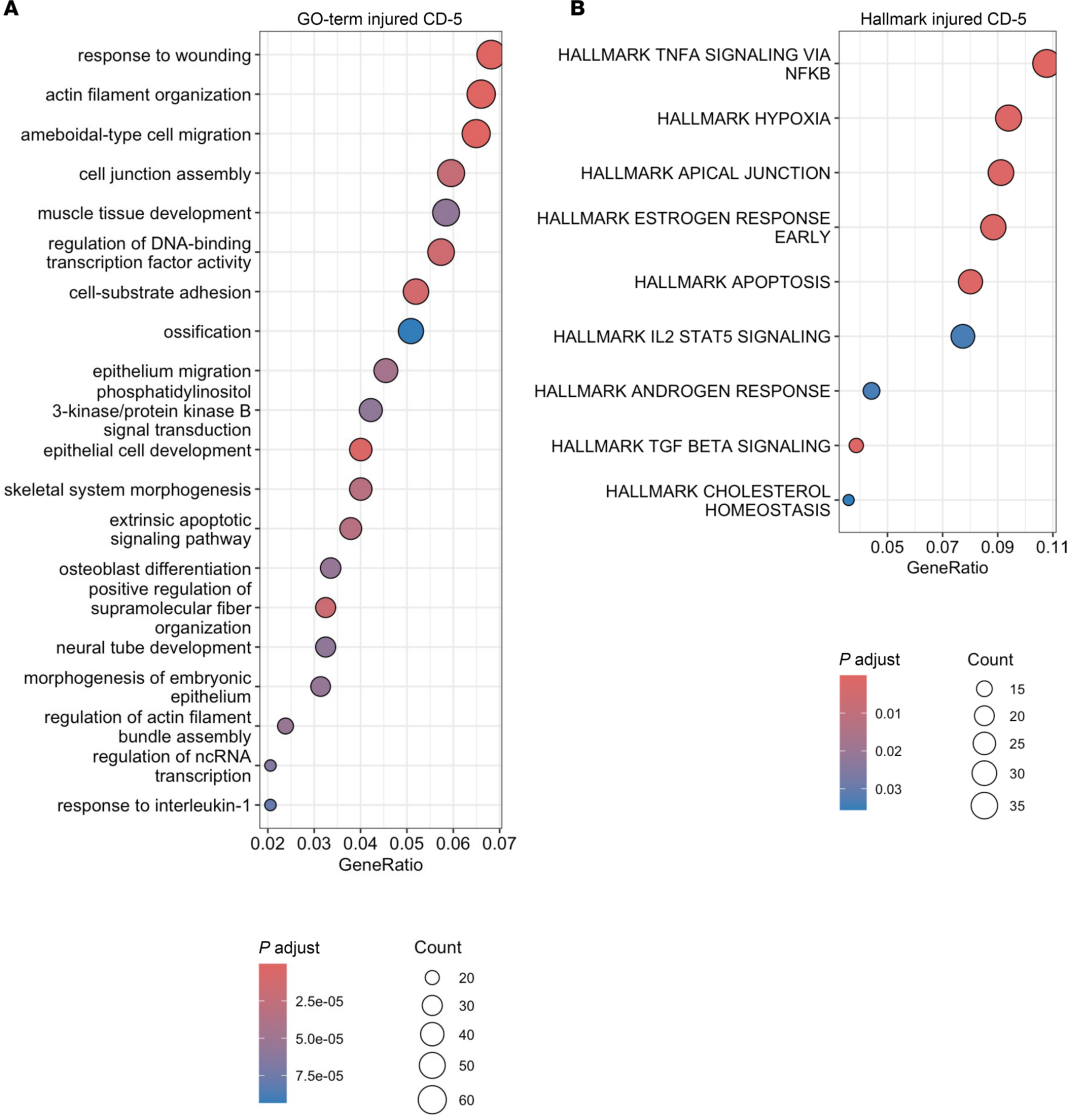
Injured inner medullary CDs are hypoxic and have features of cell migration, wounding responses, apoptosis, and inflammation. GSEA of upregulated GO terms (**A**) and Hallmark terms (**B**) in injured CD-5 DEGs.

GSEA of DEGs in injured CD-5 cells revealed enrichment for processes related to cell migration, wound healing, apoptosis, and TNFa signaling via Nfkb (Figure 9, A and B, and Supplemental Table 3). These align with injury profiles observed in injured LOH-4 cells after R-UUO and FR-PTECs after IRI-AKI and I-UUO (40). However, unlike injured LOH-4 cells and FR-PTECs, injured CD-5 cells displayed upregulation of hypoxia-related genes, including *Ldha*, *Slc2a1/Glut1*, *Pfkl*, and *Pfkp*, which drive anaerobic glycolysis and likely represent a compensatory response to hypoxia (Figure 9B, Supplemental Figure 12D, and Supplemental Table 3). DEG overlap analyses indicated that deep IM CD-0 and IM CD-2 cells also showed injury responses similar to injured CD-5 cells. Both cell types demonstrated increased cell migration, wound healing, apoptosis, inflammation, and hypoxia signatures at day 28 and 84 after R-UUO, with substantial overlap in DEGs between deep IM CD-0 and injured CD-5 cells (Supplemental Figure 19, A–C, and Supplemental Table 5). In contrast, while IM CD-2 cells exhibited hypoxia and inflammation signatures at day 28, their overlap with injured CD-5 cells diminished by day 84 (Supplemental Figure 19, F and G). These findings contrast with IM LOH clusters. Lower IM TL-5 cells displayed TNFa signaling but lacked evidence of wound healing, migration, or hypoxic responses at day 28 (Supplemental Figure 19, H and I, and Supplemental Table 6). IM ATL-0 cells showed wound healing responses at days 28 and 84 and TNF-α activation at day 28, but no hypoxia signatures were detected (Supplemental Figure 19, J and K, and Supplemental Table 7). These findings highlight widespread and persistent CD injury in the IM following R-UUO. CD injury is distinguished by a robust hypoxic response, likely reflecting adaptation to increased IM hypoxia.

### Long-term effects on cellular interactions in the renal medulla

To investigate how changes in CD, LOH, and fibroblasts affect cellular interactions in the IM after R-UUO, we analyzed ligand-receptor pairings between cell clusters (Supplemental Figure 20 and Supplemental Table 8) (66). The most notable finding was the de novo activation of the SPP1 pathway in injured LOH-4 and CD-5 cells. SPP1 signaling, mediated via integrins, promotes proinflammatory and profibrotic responses following AKI (67, 68). Other pathways that were affected included autocrine canonical Wnt signaling, which was observed in deep IM CD-0 cells, which express *Wnt7b* and *9b*. These ligands were also expressed by injured LOH-4 and injured CD-5 cells after R-UUO. PdgfA signaling showed dynamic changes: in controls, *PdgfA* was restricted to IM ATL-0 cells, with its receptors (*PdgfRA*, *PdgfRB*) found in IM Fib-2 and Fib-6 clusters. After R-UUO, *PdgfA* expression shifted to injured LOH-4 cells at day 28, with receptors primarily in Myofib-0 cells. By 84 days, *PdgfA* expression returned to IM ATL-0 cells, but Myofib-0 remained the primary responder cells. Noncanonical Wnt signaling also showed altered cellular interactions. *Wnt5A*, secreted by Fib-1 and Fib-2 clusters in controls, targeted deep IM CD-0 responder cells. After R-UUO, responder cells shifted to Myofib-0 and injured LOH-4 clusters. These findings suggest that long-term activation of these pathways contributes to the inflammatory environment and promotes remodeling between days 28 and 84 after R-UUO. These altered interactions may disrupt normal tissue homeostasis, perpetuating cellular disorganization in the IM after R-UUO.

### Common pathways are activated in IMs of patients with renal stone disease

To assess whether similar pathways are activated in patients with other IM diseases, we analyzed published snRNA-Seq data of renal papillary biopsies from patients with recurrent renal stone disease (69). Control samples were obtained from Nx patients or deceased donor kidneys unsuitable for transplantation. Two renal papillary principal cell populations, PapPC1 and PapPC2, were identified, resembling deep IM CD-0 cells in our studies. Significant overlap was found between DEGs in PapPC1 and -2 cells from patients with renal stone disease and DEGs in injured CD-5 and deep IM CD-0 cells 84 days after R-UUO (Supplemental Figure 21, A and B, and Supplemental Figure 22, A and B). GSEA showed enrichment for pathways related to wound healing, cell migration, apoptosis, and TNFa signaling in PapPC1 and -2 cells, consistent with findings in injured CD-5 and deep IM CD-0 cells after R-UUO (Supplemental Figure 21, C and D; Supplemental Figure 22, C and D; and Supplemental Tables 9 and 10). Notably, hypoxic pathway activation, a hallmark of IM CD injury following R-UUO, was absent in patients with renal stone disease. These findings indicate that shared pro-inflammatory, wound healing, and cell migration responses are activated in PapPC cells from patients with renal stone disease and IM CD cells after R-UUO. However, the hypoxic response observed in IM CD cells after R-UUO is not seen in patients with renal stone disease.

## Discussion

We present a robust model of R-UUO that allowed us to assess renal function for up to 3 months after reversing prolonged UUO by removing the contralateral kidney. Our findings demonstrate that, despite reversal of the obstruction, there is a permanent reduction in GFR accompanied by a disproportionately severe loss of UCC, indicating persistent renal medullary dysfunction. These results align with clinical observations that, beyond the acute polyuria commonly seen in the days following obstruction reversal (70, 71), some patients experience a lasting defect in their ability to concentrate urine maximally (7). This study has clinical significance for several reasons.

The renal medulla’s cellular composition and structure are conserved between rodents and humans, suggesting that the pathophysiological changes observed in this model likely reflect similar processes in humans.

The high penetrance of this phenotype in this R-UUO model indicates that such outcomes may be more common than generally recognized in clinical practice.

Impaired urine concentration increases the risk of dehydration and recurrent AKI, potentially contributing to the progressive decline in GFR observed in affected patients after reversal of urinary obstruction (5, 6).

**Figure 10:**
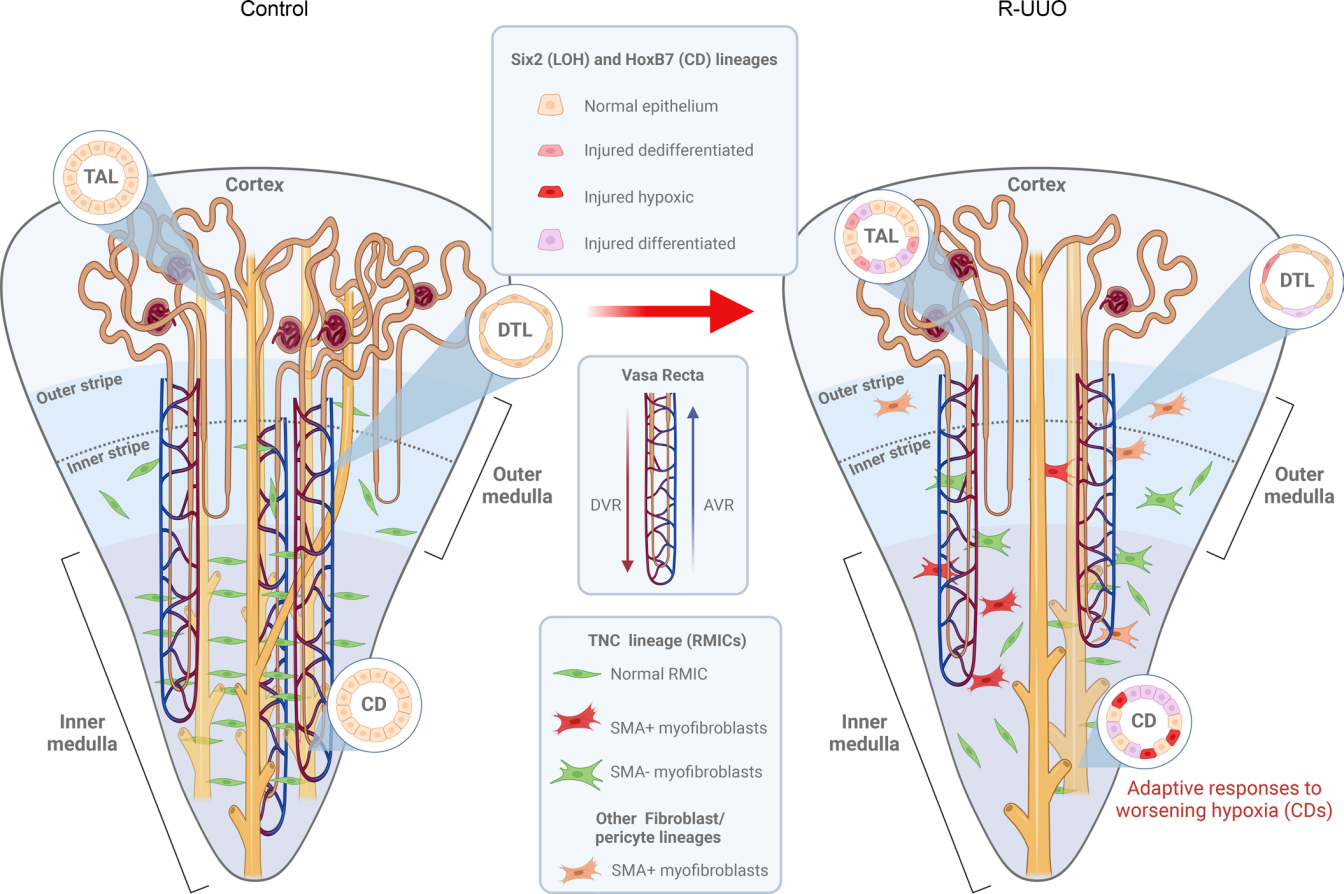
Permanent cellular defects in the renal medulla after reversal of ureteric obstruction. Summary illustrating persistent reductions in inner medullary LOH and vasa recta, decreased numbers of enlarged CDs, and depletion/mislocalization of RMICs 84 days after R-UUO. DTL, descending thin limb; DVR, descending vasa recta; AVR, ascending vasa recta.

Our studies show that while there was widespread cellular damage throughout the renal medulla along with shortening of the IM at early time points after R-UUO, there was a robust regenerative response so that by 84 days mice had normal-sized IMs that were indistinguishable from controls using standard histological stains. This regenerative response appeared to be driven by proliferation of Sox9^+^ and Ki67^+^ dedifferentiated tubular cells that repopulate denuded basement membranes. However, our scRNA-Seq and quantitative immunostaining analyses revealed that repair was incomplete, since there were permanent changes to all the major cellular compartments in the IM (illustrated in Figure 10). This included a permanent reduction in long LOH and in vasa recta and an increase in IM CD cross-sectional diameters, which could reflect a compensatory response to increased urine flow through fewer CDs after R-UUO. Despite restoration of AQP2 expression by immunostaining after R-UUO, more detailed analysis of CD clusters in the scRNA-Seq dataset showed reduced expression of water channels *Aqp2* and *3*, and the urea transporter, *Slc12a2*, in IM CD cells, which are required for the countercurrent multiplier system to concentrate urine (1). Additionally, there was a permanent reduction and mislocalization of RMICs, specialized fibroblasts critical for regulating urinary concentrating capacity through AQP2 activity in CDs (48, 49). Moreover, analysis of ligand-receptor pairing indicates that there was long-term disruption of the normal cell-cell interactions after R-UUO, including Pdgf and noncanonical Wnt signaling, which regulate both epithelial and fibroblast stability and function and could play a role in mediating tissue remodeling that occurs after R-UUO. On this basis, breakdown in the organization of all the major cellular compartments in the IM after R-UUO would be expected not only to disrupt the countercurrent multiplier system that is required to generate osmotic gradients in the renal medulla (1, 9) but also to play a role in destabilizing cellular interactions and perpetuating the cellular disorganization that occurs in the IM after R-UUO.

Following R-UUO, injured IM LOH and CD cells exhibited widespread activation of pathways regulating cell migration and inflammation. These responses mirror those seen in maladaptive or FR-PTECs, which contribute to local inflammation, fibrosis, and CKD progression after AKI (35, 36). Supporting this, ligand-receptor analysis revealed persistent de novo activation of the SPP1 pathway in injured LOH and CD cells. Since SPP1 signals through integrins to induce pro-inflammatory and pro-fibrotic responses after AKI (67, 68), this pathway likely perpetuates the pro-inflammatory state in IM epithelial cells. Interestingly, similar cell migration and pro-inflammatory pathways activated in the deep IM CD cells after R-UUO are also observed in renal papillary CDs from patients with renal stone disease (69). This suggests that these pathways represent shared epithelial responses to diverse cortical and medullary injuries in both humans and mice. For example, Mmp7, expressed by injured CDs after R-UUO, is also upregulated in papillary CDs of patients with renal stone disease (69). Furthermore, urinary MMP7 levels correlate with disease activity in renal stone disease and predict renal outcomes in children after reversal of uretero-pelvic junction obstruction (72). However, there are key differences in cellular responses to distinct types of injury. Unlike FR-PTECs after IRI-AKI and I-UUO and injured LOH cells after R-UUO, IM CDs uniquely exhibited increased expression of genes promoting anaerobic glycolysis as a compensatory response to hypoxia. This is likely to be in response to reduced oxygen supply to the IM resulting from rarefaction of vasa recta following prolonged UUO. This hypoxic adaptation enables IM CDs to survive in the increasingly hostile environment of the IM after R-UUO, whereas long LOH cells, lacking such responses, are less resilient. In contrast, the Canela et al. 2023 manuscript does not describe EC loss or capillary rarefaction in papillas from patients with renal stone disease (69). The fact that hypoxic adaptation does not occur in papillary CDs from these patients suggests that capillary rarefaction may be exerting additional long-term IM hypoxic stress after R-UUO and that this does not occur in patients with renal stone disease.

In conclusion, our findings demonstrate that despite an early, robust regenerative response after R-UUO, permanent alterations occur in all major IM cellular compartments, leading to lasting defects in UCC. These results have important implications for the management and investigation of patients with urinary obstruction.

Three months after R-UUO, the renal medulla appears histologically normal, suggesting that standard histological assessment of patients may not reveal significant changes in cellular organization after reversal of obstruction.

Focused molecular studies in patients are needed to identify common pathways that will inform the development of targeted therapies to prevent long-term renal medullary dysfunction after reversal of urinary obstruction. Notably, some molecular changes observed in this mouse model align with findings in patients with renal stone disease, another cause of renal medullary injury. This suggests the mouse model of R-UUO reflects similar pathophysiological changes occurring in humans, providing valuable insights into potential therapeutic strategies.

## Methods

### Sex as a biological variable.

Our studies only evaluated male mice. It is currently unknown whether these findings are relevant to female mice.

### Mouse lines and strains.

Male BALB/c mice were purchased from Charles River. HoxB7-Cre–transgenic mice [*Tg(HoxB7-Cre)13Amc*] (73) and *Six2* *eGFP-Cre BAC*–transgenic mice [*Tg(Six2-EGFP/Cre)1Amc/J*] (74) were provided by Andy McMahon from Keck School of Medicine of University of Southern California, Los Angeles, California, USA. tdTomato-Cre reporter mice and *Tenascin C Cre-ERT2-IRES-GFP*–knockin mice (64) were provided by Agnes Fogo from Vanderbilt University Medical Center with permission from Chuan-Ming Hao from Fudan University in Shanghai, China. *B6.Cg-Gt(ROSA)26Sor^tm14(CAG-tdTomato)Hze^*/J mice were obtained from Jackson Laboratory. Six2- and HoxB7-Cre mice were on mixed C57BL/6 CD-1/Swiss Webster backgrounds, and *Tenascin C Cre-ERT2-IRES-GFP*–knockin and tdTomato-Cre reporter mouse lines were on a C57BL/6 background. Mice were genotyped and identified from punch biopsies using allele-specific primers performed by Transnetyx. Genotyping primers are listed in Supplemental Table 1.

### R-UUO.

R-UUO was performed in 12- to 13-week-old male mice. For this, a small vascular clamp was applied to the left ureter, and the whole kidney with the ureteral clamp in place was gently pushed back into the retroperitoneal space. After a variable interval of time depending on the mouse strain (between 5 and 7 days), the left kidney was exteriorized, and the vascular clamp was carefully removed. For long-term studies, a contralateral Nx was performed 10 days after R-UUO. Nx controls were performed at the same age/time point as the R-UUO studies. After completion of the studies, the left kidney was initially checked to confirm that there was reversal of the obstruction, and if uncertain, methylene blue was injected into the renal pelvis to determine whether the left ureter was patent. Since external evidence of hydronephrosis resolves within approximately 24 hours of reversal of obstruction, if there was evidence of persistent obstruction, data were discarded (Supplemental Methods 1 includes detailed information about tissue harvesting, surgical technique, and renal function tests).

### Immunofluorescence staining.

Formalin-fixed and frozen and FFPE sections were prepared, washed, and blocked, and primary and secondary antibodies were applied, as described (see Supplemental Table 1 for the list of antibodies, fluorophores, and conditions) (54). Digital images were scanned using AxioScan Z1 slide scanner (Carl Zeiss Microscopy GmbH; Original magnification, 10×), then downloaded into QuPath for analysis (Version 0.5.0). For quantification, regions of interest (cortex, OSOM, ISOM, proximal third and distal third of the IM) were demarcated using the QuPath annotation tool. If sections through the IM were less than 1 mm in length, we would quantify only the area of interest in the proximal IM. To quantify CD surface areas, digital images of HoxB7 lineage–labeled sections with IM lengths of ≥1 mm were used. To quantify tubular surface areas, we evaluated circular CDs defined as having major/minor axis ratios of >0.8, as described (75). We quantified the surface area of each tubule and its lumen using the freehand selection tool in Fiji: 15 and 30 tubules in each area of interest and the number of nuclei counted in each tubule cross section (Supplemental Methods 1 includes information on staining and image quantification, and Supplemental Methods 2 provides a detailed immunofluorescence staining protocol).

### scRNA-Seq.

Renal medullas were dissected at 2 time points after injury (day 28, 84 after R-UUO) and from healthy controls. Three to 4 renal medullas from the same time point were placed in a cryogenic vial and flash-frozen with liquid nitrogen, then stored at –80°C. We evaluated 4 of these pooled samples from healthy controls, 4 from mice at day 28, and 2 from dat 84 after R-UUO. Each frozen tube was fixed and dissociated into single cells using the 10x Genomics Chromium Next GEM Single Cell Fixed RNA Sample Preparation Kit 1000414. Dissociated cells from each of the pooled samples were individually hybridized with bar-coded genome-wide probe sets from 10x Genomics Flex kits according to the manufacturer’s instructions. After probe hybridization and construction of libraries, samples were sequenced using an Illumina NovaSeq 6000 PE150 sequencer (see Supplemental Methods 3 for a detailed sample preparation protocol).

### Bioinformatics pipeline and analysis of transcriptome data.

After quality control measures were applied, dimensionality reduction was performed using principal component analysis and visualized with UMAP. Cell population identities were identified using published anchor genes for renal medullary LOH, CD, fibroblast, EC, and immune cell subclusters (44, 52, 53). Differential gene expression analysis between clusters and time points was carried out using the FindAllMarkers function and the FindMarkers function, respectively. GO and Hallmark pathway GSEA was performed and visualized by ClusterProfiler, as well as published FR-PTEC DEG gene sets from published data (40). The subset function was used to extract clusters of each cell type on which principal component analysis, reclustering, and differential gene expression analysis were carried out. The injured LOH cluster 4 was subclustered using the FindSubCluster function. Cell-cell communication analysis was performed using CellChat (66). Data were visualized using Seurat, dittoSeq, and ShinyCell R packages (see Supplemental Methods 1 for additional information about the quality control and analytical approaches used to evaluate the scRNA-Seq data) (76).

### Manuscript editing.

We used generative AI (ChatGPT 4.0) to edit the text during revision of this manuscript. We used ChatGPT between December 1, and December 5, 2024, submitting all of the draft paragraphs or figure legends we had written with the following prompt: “please rewrite this text (or Figure legend) so that it is easier to read but is more concise.”

### Statistics.

Statistics were performed using GraphPad Prism 10.1.2 software, and results expressed as means ± SEM, with individual data points shown where indicated. Two-tailed, unpaired *t* tests were used to compare 2 groups; 1-way ANOVA to compare between 3 or more groups; and 2-way ANOVA to compare groups over time. If *P* values were less than 0.05 for between-group analyses, multiple between-group comparisons were performed controlling for false discovery rates of *P* < 0.05 using the 2-step method of Benjamini, Krieger, and Yekutieli. *q* values are indicated after correction for multiple between-group comparisons. Statistical significance between multiset intersections from the scRNA-Seq subcluster Venn diagrams was performed using the SuperExactTest function in R (77).

### Study approval.

All mouse experiments were approved by the Vanderbilt University Medical Center Institutional Animal Care and Use Committee.

### Data availability.

All relevant data are found in this article and in the Supporting Data Values file. scRNA-Seq data have been deposited in the NCBI’s Gene Expression Omnibus repository: Study ID: GSE283241.

## Author contributions

TV performed the scRNA-Seq data analysis and prepared the scRNA-Seq figures and supplemental datasets with help from AG. RD performed mouse genotyping, R-UUO surgery, and renal function assays with help from MB, KAC, and AAM. MB, AAM, WES, TR, and VS performed immunostaining and quantitative image analyses. RD quantified SR staining. HY provided renal pathology advice on R-UUO samples. With help from AJD, MPDC conceptualized and planned studies, interpreted all the data, finalized and prepared figures, and wrote the manuscript.

## Supplementary Material

Supplemental data

Supplemental table 1

Supplemental table 10

Supplemental table 11

Supplemental table 2

Supplemental table 3

Supplemental table 4

Supplemental table 5

Supplemental table 6

Supplemental table 7

Supplemental table 8

Supplemental table 9

Supporting data values

## Version 1 01/23/2025

In-Press Preview

## Version 2 03/10/2025

Electronic publication

## References

[1] Sands JM, Layton HE (2014). Advances in understanding the urine-concentrating mechanism. Annu Rev Physiol.

[2] Nawata CM, Pannabecker TL (2018). Mammalian urine concentration: a review of renal medullary architecture and membrane transporters. J Comp Physiol B.

[3] Tseng TY, Stoller ML (2009). Obstructive uropathy. Clin Geriatr Med.

[4] Wu AK (2012). Relative renal function does not improve after relieving chronic renal obstruction. BJU Int.

[5] Caddeo G (2013). Acute kidney injury in urology patients: incidence, causes and outcomes. Nephrourol Mon.

[6] Sasaki S (2017). Anemia and long-term renal prognosis in patients with post-renal acute kidney injury of nonmalignant cause. Nephron.

[7] Berlyne GM (1961). Distal tubular function in chronic hydronephrosis. Q J Med.

[8] (1956). Effects of complete ureteral obstruction in dogs on kidney function. Am J Physiol.

[9] Dantzler WH (2011). Urine concentrating mechanism in the inner medulla of the mammalian kidney: role of three-dimensional architecture. Acta Physiol (Oxf).

[10] Klahr S (1983). Pathophysiology of obstructive nephropathy. Kidney Int.

[11] Wilson DR (1977). Renal function during and following obstruction. Annu Rev Med.

[12] Zhang W, Edwards A (2002). Oxygen transport across vasa recta in the renal medulla. Am J Physiol Heart Circ Physiol.

[13] Griffin MD (1995). Renal papillary necrosis--a sixteen-year clinical experience. J Am Soc Nephrol.

[14] Onen A (2020). Grading of hydronephrosis: an ongoing challenge. Front Pediatr.

[15] Hiatt MJ (2013). Urinary tract obstruction in the mouse: the kinetics of distal nephron injury. Lab Invest.

[16] Wilson DR (1972). Micropuncture study of chronic obstructive nephropathy before and after release of obstruction. Kidney Int.

[17] Wei G (2015). Architecture of the human renal inner medulla and functional implications. Am J Physiol Renal Physiol.

[18] Bander SJ (1985). Long-term effects of 24-hr unilateral ureteral obstruction on renal function in the rat. Kidney Int.

[19] Li C (2001). Downregulation of AQP1, -2, and -3 after ureteral obstruction is associated with a long-term urine-concentrating defect. Am J Physiol Renal Physiol.

[20] Puri TS (2010). Chronic kidney disease induced in mice by reversible unilateral ureteral obstruction is dependent on genetic background. Am J Physiol Renal Physiol.

[21] Buchtler S (2018). Cellular origin and functional relevance of collagen I production in the kidney. J Am Soc Nephrol.

[22] Tapmeier TT (2008). Reimplantation of the ureter after unilateral ureteral obstruction provides a model that allows functional evaluation. Kidney Int.

[23] Conway BR (2020). Kidney single-cell atlas reveals myeloid heterogeneity in progression and regression of kidney disease. J Am Soc Nephrol.

[24] Connor KL (2020). Identifying cell-enriched miRNAs in kidney injury and repair. JCI Insight.

[25] Hesketh EE (2014). A murine model of irreversible and reversible unilateral ureteric obstruction. J Vis Exp.

[26] Yao Y (2011). Interferon-γ improves renal interstitial fibrosis and decreases intrarenal vascular resistance of hydronephrosis in an animal model. Urology.

[27] Chaabane W (2013). Renal functional decline and glomerulotubular injury are arrested but not restored by release of unilateral ureteral obstruction (UUO). Am J Physiol Renal Physiol.

[28] Cochrane AL (2005). Renal structural and functional repair in a mouse model of reversal of ureteral obstruction. J Am Soc Nephrol.

[29] Souma T (2013). Plasticity of renal erythropoietin-producing cells governs fibrosis. J Am Soc Nephrol.

[30] Flam T (1984). Reversible hydronephrosis in the rat: a new surgical technique assessed by radioisotopic measurements. J Urol.

[31] Hammad FT (2020). Despite initial recovery of GFR, long-term renal functions deteriorate following short periods of unilateral ureteral obstruction. Am J Physiol Renal Physiol.

[32] Hammad FT, Lubbad L (2011). The effect of diclofenac sodium on renal function in reversible unilateral ureteric obstruction. Urol Res.

[33] Ito K (2004). Renal damage progresses despite improvement of renal function after relief of unilateral ureteral obstruction in adult rats. Am J Physiol Renal Physiol.

[34] Hinze C (2024). Epithelial cell states associated with kidney and allograft injury. Nat Rev Nephrol.

[35] Kirita Y (2020). Cell profiling of mouse acute kidney injury reveals conserved cellular responses to injury. Proc Natl Acad Sci U S A.

[36] Gerhardt LMS (2021). Single-nuclear transcriptomics reveals diversity of proximal tubule cell states in a dynamic response to acute kidney injury. Proc Natl Acad Sci U S A.

[37] Ledru N (2024). Predicting proximal tubule failed repair drivers through regularized regression analysis of single cell multiomic sequencing. Nat Commun.

[38] Lake BB (2023). An atlas of healthy and injured cell states and niches in the human kidney. Nature.

[39] Hinze C (2022). Single-cell transcriptomics reveals common epithelial response patterns in human acute kidney injury. Genome Med.

[40] Li H (2022). Comprehensive single-cell transcriptional profiling defines shared and unique epithelial injury responses during kidney fibrosis. Cell Metab.

[41] Gerhardt LMS (2023). Lineage tracing and single-nucleus multiomics reveal novel features of adaptive and maladaptive repair after acute kidney injury. J Am Soc Nephrol.

[42] Gerhardt LMS, McMahon AP (2022). Identifying common molecular mechanisms in experimental and human acute kidney injury. Semin Nephrol.

[43] Muto Y (2024). Epigenetic reprogramming driving successful and failed repair in acute kidney injury. Sci Adv.

[44] Hinze C (2021). Kidney single-cell transcriptomes predict spatial corticomedullary gene expression and tissue osmolality gradients. J Am Soc Nephrol.

[45] Neuhofer W, Beck FX (2006). Survival in hostile environments: strategies of renal medullary cells. Physiology (bethesda).

[46] Bulger RE, Trump BF (1966). Fine structure of the rat renal papilla. Am J Anat.

[47] Hughes AK, Kohan DE (2006). Mechanism of vasopressin-induced contraction of renal medullary interstitial cells. Nephron Physiol.

[48] Zhang MZ (2018). Renal medullary interstitial COX-2 (cyclooxygenase-2) is essential in preventing salt-sensitive hypertension and maintaining renal inner medulla/papilla structural integrity. Hypertension.

[49] Hu C (2020). Renomedullary interstitial cell endothelin a receptors regulate BP and renal function. J Am Soc Nephrol.

[50] Galarreta CI (2014). Tubular obstruction leads to progressive proximal tubular injury and atubular glomeruli in polycystic kidney disease. Am J Pathol.

[51] Wu H (2019). Advantages of single-nucleus over single-cell RNA sequencing of adult kidney: rare cell types and novel cell states revealed in fibrosis. J Am Soc Nephrol.

[52] Ransick A (2019). Single-cell profiling reveals sex, lineage, and regional diversity in the mouse kidney. Dev Cell.

[53] Dumas SJ (2020). Single-cell RNA sequencing reveals renal endothelium heterogeneity and metabolic adaptation to water deprivation. J Am Soc Nephrol.

[54] Yang M (2023). Inhibition of retinoic acid signaling in proximal tubular epithelial cells protects against acute kidney injury. JCI Insight.

[55] Jablonski KA (2015). Novel markers to delineate murine M1 and M2 macrophages. PLoS One.

[56] Lever JM (2019). Resident macrophages reprogram toward a developmental state after acute kidney injury. JCI Insight.

[57] England AR (2020). Identification and characterization of cellular heterogeneity within the developing renal interstitium. Development.

[58] Xin C (2021). Therapeutic silencing of SMOC2 prevents kidney function loss in mouse model of chronic kidney disease. iScience.

[59] Kwon MS (2009). Hypertonic stress in the kidney: a necessary evil. Physiology (bethesda).

[60] Zhen YY (2022). Coordination of LMO7 with FAK signaling sustains epithelial integrity in renal epithelia exposed to osmotic pressure. Cells.

[61] Kramann R (2015). Perivascular Gli1+ progenitors are key contributors to injury-induced organ fibrosis. Cell Stem Cell.

[62] Kramann R (2017). Gli1^+^ pericyte loss induces capillary rarefaction and proximal tubular injury. J Am Soc Nephrol.

[63] Kuppe C (2021). Decoding myofibroblast origins in human kidney fibrosis. Nature.

[64] He W (2013). Generation of a tenascin-C-CreER2 knockin mouse line for conditional DNA recombination in renal medullary interstitial cells. PLoS One.

[65] Neelisetty S (2015). Renal fibrosis is not reduced by blocking transforming growth factor-β signaling in matrix-producing interstitial cells. Kidney Int.

[66] Jin S (2021). Inference and analysis of cell-cell communication using CellChat. Nat Commun.

[67] Khamissi FZ (2022). Identification of kidney injury released circulating osteopontin as causal agent of respiratory failure. Sci Adv.

[68] Ding H (2024). Kidney fibrosis molecular mechanisms Spp1 influences fibroblast activity through transforming growth factor beta smad signaling. iScience.

[69] Canela VH (2023). A spatially anchored transcriptomic atlas of the human kidney papilla identifies significant immune injury in patients with stone disease. Nat Commun.

[70] Leinum LR (2020). Post-obstructive diuresis; underlying causes and hospitalization. Scand J Urol.

[71] Khara S (2019). Post-obstructive diuresis: a complication of urinary retention. Br J Hosp Med (Lond).

[72] Wang HS (2022). Association between urinary biomarkers MMP-7/TIMP-2 and reduced renal function in children with ureteropelvic junction obstruction. PLoS One.

[73] Yu J (2002). Sonic hedgehog regulates proliferation and differentiation of mesenchymal cells in the mouse metanephric kidney. Development.

[74] Kobayashi A (2008). Six2 defines and regulates a multipotent self-renewing nephron progenitor population throughout mammalian kidney development. Cell Stem Cell.

[75] Uehara-Watanabe N (2022). Direct evidence of proximal tubular proliferation in early diabetic nephropathy. Sci Rep.

[76] Bunis DG (2021). dittoSeq: universal user-friendly single-cell and bulk RNA sequencing visualization toolkit. Bioinformatics.

[77] Wang M (2015). Efficient test and visualization of multi-set intersections. Sci Rep.

